# SLIPPERS Reconsidered: Clinical, Radiological, and Pathological Overlap with PACNS—A Case Report

**DOI:** 10.3390/reports9010047

**Published:** 2026-01-31

**Authors:** Inhar Esnaola Barriola, Celia Fernández Gonzalez, Teresa Cabada Giadas, María Victoria Zelaya Huerta, María Elena Erro Aguirre

**Affiliations:** 1 Neurology Department, University Hospital of Navarra, 31008 Pamplona, Spain; elena.erro.aguirre@navarra.es; 2 Radiology Department, University Hospital of Navarra, 31008 Pamplona, Spain; celia.fernandez.gonzalez@navarra.es (C.F.G.); teresa.cabada.giadas@navarra.es (T.C.G.); 3 Pathology Department, University Hospital of Navarra, 31008 Pamplona, Spain; mv.zelaya.huerta@navarra.es

**Keywords:** primary angiitis of the central nervous system, SLIPPERS syndrome, magnetic resonance imaging, brain biopsy, immunosuppressive therapy, case report

## Abstract

Background and Clinical Significance: SLIPPERS syndrome (Supratentorial Lymphocytic Inflammation with Parenchymal Perivascular Enhancement Responsive to Steroids) was first described in 2015 as a variant of CLIPPERS restricted to supratentorial regions. Only a few cases have been reported so far, and its distinction from primary angiitis of the central nervous system (PACNS) remains challenging, as both may present with overlapping clinical, radiological, and histopathological features. We report two patients initially diagnosed with SLIPPERS but finally fulfilling the diagnostic criteria for PACNS, highlighting the complexity of the differential diagnosis. Case Presentation: The first patient was a 49-year-old woman who presented with seizures, memory impairment, and facial neuralgia. MRI showed multiple cortico-subcortical and deep nodular lesions in the left hemisphere with gadolinium enhancement. Brain biopsy revealed a T-cell-predominant lymphocytic vascular infiltrate. She responded to corticosteroids but later relapsed, requiring methotrexate for long-term immunosuppression, with no further recurrences during seven years of follow-up. The second patient was a 64-year-old man with hypertension, dyslipidemia, and alcohol use who developed repeated focal-to-generalized seizures. MRI disclosed multifocal nodular gadolinium-enhancing right hemispheric lesions, with SWI microhemorrhages. Biopsy demonstrated transmural T-cell vasculitic infiltrates. He responded to corticosteroids and methotrexate, but radiological progression at 14 months prompted replacement with cyclophosphamide. Conclusions: There is a considerable clinical, radiological, and histological overlap between SLIPPERS and PACNS. Careful analysis of advanced MRI sequences, particularly angiographic and vessel-wall imaging studies, combined with meticulous histopathological analysis, is essential to avoid misdiagnosis. These similarities suggest that some cases attributed to SLIPPERS may, in fact, correspond to variants of PACNS.

## 1. Introduction and Clinical Significance

SLIPPERS syndrome (Supratentorial Lymphocytic Inflammation with Parenchymal Perivascular Enhancement Responsive to Steroids), a term introduced in 2015, five years after the description of CLIPPERS (Chronic Lymphocytic Inflammation with Pontine Perivascular Enhancement Responsive to Steroids), defines a clinically and pathologically similar entity that differs in the distribution of lesions. The hallmark of SLIPPERS is the presence of multifocal supratentorial lesions that exhibit a characteristic pseudonodular gadolinium enhancement pattern on MRI and resolution following glucocorticoid administration. Histopathologically, the syndrome is defined by a dense perivascular T-cell-predominant lymphocytic infiltration [1]. To date, only eleven cases have been reported in the literature. The differential diagnosis of SLIPPERS is broad and includes primary angiitis of the central nervous system (PACNS), which itself represents a diagnostic challenge due to the heterogeneity of its radiological features and the difficulties inherent to pathological confirmation [2,3]. The aim of this report is to review the published cases of SLIPPERS in light of two patients who, although fulfilling the proposed diagnostic criteria for this syndrome, were ultimately confirmed as having PACNS after a reassessment of their radiological and pathological findings.

## 2. Case Presentation

Both patients were clinically evaluated by neurologists from the Department of Neurology at the University Hospital of Navarra. Their medical records, anatomopathological study, and magnetic resonance imaging (MRI) were reviewed. This study was conducted in accordance with the ethical standards of our institution, and written informed consent was obtained from both participants.

### 2.1. Case 1

#### 2.1.1. Initial Presentation

A 49-year-old woman with a history of smoking and no other significant underlying medical conditions was admitted in-hospital following a seizure. In the preceding months, she had experienced facial neuralgia and memory lapses. Neurological examination and electroencephalography (EEG) were normal.

#### 2.1.2. Investigations

**Figure 1:**
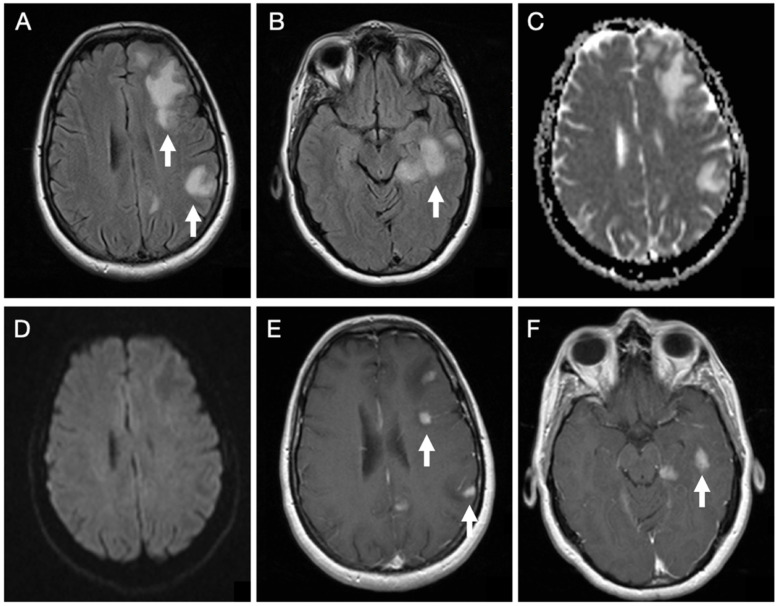
Initial brain MRI on axial FLAIR sequences of patient 1 demonstrates several hyperintense foci within the subcortical white matter of the left frontal, parietal, and temporal lobes (**A**,**B**). Diffusion-weighted imaging in the B1000 sequence and the corresponding ADC map reveal facilitated diffusivity of the lesions (**C**,**D**). Post-contrast T1-weighted images show multiple nodular enhancing foci (**E**,**F**).

Brain MRI (Figure 1) revealed cortico-subcortical lesions in the left hemisphere involving the frontal, parietal, and temporal lobes, in addition to deep lesions in the corona radiata and posterior thalamus. These appeared hyperintense on T2-weighted spin-echo sequence and Fluid Attenuated Inversion Recovery (FLAIR) sequences, with associated perilesional edema, showing facilitated diffusion, except for a single lesion in the corona radiata that demonstrated restricted diffusion. The lesions showed an intense enhancing nodular pattern after gadolinium administration.

Systemic evaluation was unrevealing, including chest and abdominal CT, whole-body PET, serologies, tumor markers, and autoimmune panels.

Cerebrospinal fluid (CSF) analysis revealed a white blood cell count of 3 cells/mm^3^ and a red blood cell count of 250 cells/mm^3^. Biochemistry showed normal levels of glucose (66 mg/dL) and protein (26 mg/dL). Microbiological screening, including Gram stain, bacterial culture, and PCR for Enterovirus, HSV-1, HSV-2, and VZV, was negative. Cytological examination showed no evidence of neoplastic cells. Immunological studies confirmed the absence of oligoclonal bands (OCBs) and no evidence of intrathecal IgG synthesis (IgG: 1.22 mg/dL; IgM: <0.21 mg/dL). The albumin quotient was within normal limits. An extensive paraneoplastic and autoimmune panel was performed at a reference laboratory. Testing was negative for onconeural antibodies (anti-Tr/DNER, GAD65, Zic4, SOX1, Hu, Yo, Ri, Ma1, Ma2, CV2/CRMP5, and Amphiphysin). Additionally, cell-surface antibody testing (anti-NMDAR, AMPAR, GABAbR, DPPX, IgLON5, LGI1, and CASPR2) using rat brain tissue via the avidin-biotin-peroxidase technique yielded no pathological immunoreactivity (Table 1).

#### 2.1.3. Histopathology

**Figure 2:**
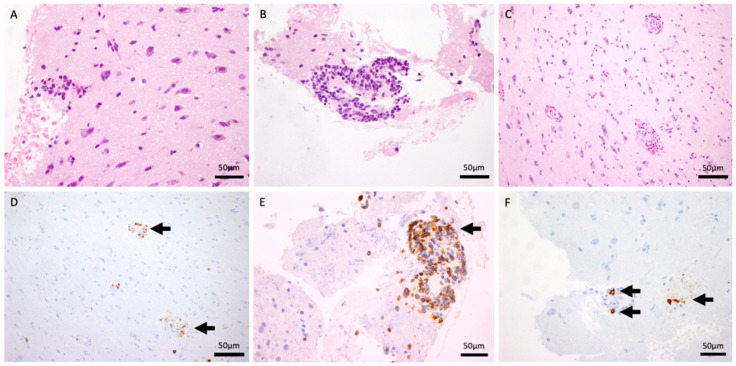
H&E stain (40×) demonstrating intense chronic perivascular inflammatory infiltrates within the meninges and cortex (**A**,**B**). H&E stain (20×) showing evidence of slight erythrocyte extravasation (**C**). CD20 immunohistochemistry (20×) revealing isolated B-cell presence (**D**). T-cell markers (CD3, CD5, and CD8, 40×) confirming an intense T-cell-predominant infiltrate (**E**). CD138 stain (40×) highlighting occasional reactive plasma cells (**F**).

An open brain biopsy (Figure 2) was performed twice, as the initial procedure was inconclusive. Histological analysis demonstrated fragments of the meninges with intense chronic inflammation, in continuity with the cerebral cortex and a small fragment of white matter. A preserved neuronal and glial population was present (A: HE 40×). No tumor proliferation was observed. There was a perivascular inflammatory infiltrate without evidence of atypical lymphocytes. These were mostly small, reactive lymphocytes with occasional plasma cells (B: HE and F: CD138, 40×). There was evidence of slight erythrocyte extravasation (C: HE 20×). Immunohistochemical analysis demonstrates isolated positivity for CD20 (D: 20×) and intense positivity for CD3, CD5, and CD8 (E: 40×). No parasites or fungi were identified. Epstein–Barr virus, herpes simplex virus type 8, and CMV were negative. A vascular elastic stain was not performed during the initial workup, as the clinical suspicion was focused on the perivascular inflammation. Subsequent attempts to obtain this stain retrospectively were precluded by the exhaustion of the tissue block. However, a meticulous re-evaluation of the existing H&E sections by an experienced neuropathologist unequivocally demonstrated the transmural nature of the infiltrate, with lymphocytes invading the full thickness of the vessel wall.

#### 2.1.4. Treatment and Outcome

In the first episode, the patient was started on intravenous dexamethasone (4 mg every 8 h) for 3 days, followed by a taper to 2 mg every 8 h for 3 days, and then 1 mg every 8 h for subsequent days before transitioning to high-dose oral therapy. The patient initially responded to corticosteroids, with subsequent clinical and radiological improvement, but seven months later, she relapsed, with enlargement of previous lesions and the appearance of new foci. She received methylprednisolone pulses (1 g/day), followed by oral prednisone at 60 mg/day. The dose was tapered by 10 mg every month, maintaining a dose of 10 mg/day during the next two years. As maintenance therapy, methotrexate was added to oral prednisone, initiated weekly with a progressive titration: 10 mg/week (weeks 1–2), 12.5 mg/week (weeks 3–4), 15 mg/week (weeks 5–6), 17.5 mg/week (weeks 7–8), and a stable maintenance dose of 20 mg/week from week 9 onwards. During a seven-year follow-up, no further relapses were observed.

### 2.2. Case 2

#### 2.2.1. Initial Presentation

A 64-year-old man with a history of hypertension, dyslipidemia, and chronic alcohol consumption, with no other significant underlying medical conditions, presented with four focal-onset generalized seizures over a six-week period. Neurological examination, EEG, and initial cranial computed tomography (CT) were normal.

#### 2.2.2. Investigations

**Figure 3:**
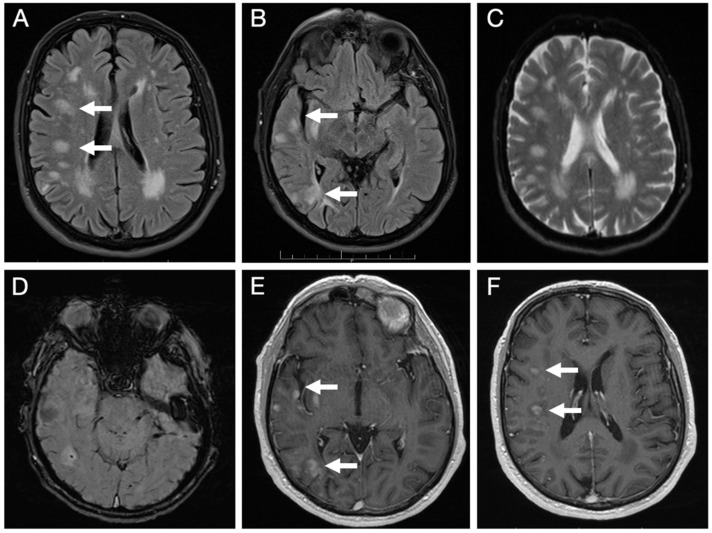
Initial brain MRI on axial FLAIR sequences shows multiple foci of hyperintense signal abnormalities in the subcortical and juxtacortical white matter of the right hemisphere (**A**,**B**); with facilitated diffusion (**C**); areas of signal loss compatible with microhemorrhages on SWI sequence (**D**); and patchy, nodular enhancement on post-contrast T1-weighted images (**E**,**F**).

Brain MRI (Figure 3) revealed multiple lesions in the right hemisphere, involving subcortical and juxtacortical white matter and the cortical regions of all lobes, as well as the lenticular nucleus. The lesions were nodular, hyperintense on T2/FLAIR with facilitated diffusion, and showed patchy contrast enhancement. SWI sequences demonstrated areas of signal loss compatible with chronic microhemorrhages. Systemic evaluation was negative.

CSF examination showed cell counts of 3 white blood cells/mm^3^ and 3 red blood cells/mm^3^, with a glucose level of 59 mg/dL. A mild elevation in total protein was noted (79 mg/dL), though the adenosine deaminase (ADA) level was normal (1 U/L). Gram stain and bacterial cultures were negative. To exclude leptomeningeal malignancy, cytology and flow cytometry were performed, both of which were negative for malignant cells or lymphoproliferative disorders. Anti-MOG antibodies in the CSF were also negative. Systemic evaluation to rule out paraneoplastic syndromes included a normal serum proteinogram (no monoclonal spikes) and a comprehensive panel of tumor markers. Serum levels of PSA, AFP, CEA, Ca 15.3, Ca 19.9, Cyfra 21.1, neuron-specific enolase (NSE), and S100 protein were all within physiological ranges (Table 1).

**Table 1:** Cerebrospinal fluid findings.

Parameter	Case 1	Case 2
White Cell Count	3 cells/mm^3^	3 cells/mm^3^
Protein/Glucose	26/66 mg/dL	79/59 mg/dL
Oligoclonal Bands/IgG Index	Negative/Normal	Not detected
Microbiology	PCR (HSV, VZV, EntV) Negative	Culture/Gram Negative
Cytology/Cytometry	Negative	Negative
Autoimmune Panel	Negative (15+ antibodies)	Anti-MOG Negative

#### 2.2.3. Histopathology

**Figure 4:**
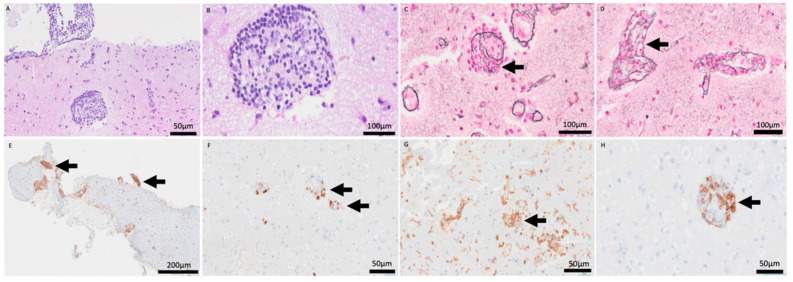
H&E stain (20×, 60×) revealing a prominent perivascular sleeve-like arrangement of inflammatory cells (**A**,**B**). Elastic stain (60×) confirming the transmural nature of the infiltrate, invading and disrupting the vascular wall (**C**,**D**). CD79a (5×) and CD20 (20×) stains showing partial B-cell lineage markers (**E**,**F**). CD4 (10×) and CD8 (20×) stains highlighting the predominance of T-lymphocytes within the vascular wall (**G**,**H**).

Open brain biopsy (Figure 4) demonstrated a cerebral parenchyma with slightly altered architecture. Astrocytosis and reactive gliosis were present. A striking inflammatory infiltrate with a perivascular sleeve-like arrangement was present (A: HE 20×), composed of small-to-medium-sized cells dispersed throughout the cerebral parenchyma (B: HE 60×). Transmural infiltration was observed with elastic staining (C–D: Elastic stain (60×).

No areas of necrosis were identified. Immunohistochemical analysis showed partial B-cell lineage markers (E: CD79a, 5×, F: CD20, 20×) with predominance of T-lymphocytes (G: CD4, 10×, H: CD8, 20×). Epstein–Barr virus was negative. B- and T-cell clonality testing was requested, and the result was positive for T-cell clonality. However, because this molecular finding did not correlate with cytological atypia or the immunohistochemical profile, the pathological diagnosis was determined to be a reactive inflammatory process.

#### 2.2.4. Treatment and Outcome

Initially, the patient received intravenous methylprednisolone pulses (1 g/day) for 4 consecutive days, followed by oral prednisone with a descending taper. As maintenance therapy, methotrexate was titrated from the outset, starting at 2.5 mg/day for 10 days, then increased to 5 mg/day. It was subsequently transitioned to a weekly regimen: 7.5 mg/week, then 10 mg/week, then 15 mg/week, and eventually reaching 25 mg/week at five months. Following radiological progression after 14 months on methotrexate, the patient received six monthly cycles of intravenous cyclophosphamide. To maintain remission after the cyclophosphamide cycles, he was transitioned to mycophenolate mofetil (500 mg every 12 h) for 3 weeks, which was then increased to a maintenance dose of 1000 mg every 12 h.

## 3. Discussion

We present two patients in whom an initial diagnosis of SLIPPERS syndrome was established due to the association of supratentorial brain lesions with response to steroids and pathological findings comparable to those described in CLIPPERS. However, both experienced clinical and radiological relapses, ultimately leading to reconsideration of the initial diagnosis in favor of PACNS. The recurrence observed in the second case prompted a comprehensive reassessment of the radiologic and pathologic findings and brought to attention the first case, which had remained in long-term remission but was re-evaluated. The possibility of other neurological entities responding to steroids was considered, such as primary lymphoma of the central nervous system, but the course of the disease, with no new recurrences in years in the first case and clinical stability in the second case, allowed us to rule out this diagnostic option.

To ensure a robust diagnosis, both cases were evaluated against the classical criteria for PACNS. According to Calabrese and Mallek [4], PACNS requires an unexplained neurological deficit, histopathologic or angiographic evidence of vasculitis, and the strict exclusion of systemic conditions. Both of our patients met these requirements (Table 2), achieving also a “Definite” diagnosis under the Birnbaum and Hellmann framework [5] due to tissue biopsy confirmation. Review of the histological samples revealed that the lymphocytic infiltrate was transmural, invading and disrupting the vascular wall rather than being exclusively perivascular. Radiological findings were also consistent with PACNS, showing multifocal cortico-subcortical lesions that were hyperintense on T2/FLAIR, nodular morphology, facilitated diffusion, microhemorrhages, and patchy contrast enhancement following gadolinium administration.

The 1988 diagnostic criteria for PACNS were published prior to the widespread availability of MRI and therefore do not incorporate radiological features. Brain MRI demonstrates abnormalities in approximately 90% of patients with PACNS [5]. The most common findings are multifocal hyperintensities on T2/FLAIR, which may correspond to acute or chronic ischemic changes [6]. These lesions are often multiple and bilateral and can involve both cortical and subcortical regions [7]. Gadolinium enhancement is another hallmark, which may be parenchymal with patchy or nodular patterns, particularly in tumefactive PACNS [6], or leptomeningeal, although the latter is nonspecific and may occur in neoplastic, infectious, or other inflammatory disorders [2]. Susceptibility-weighted imaging (SWI) and gradient echo sequences (GRE) may reveal parenchymal microhemorrhages, as well as intracranial or even subarachnoid hemorrhage [8]. Magnetic resonance angiography (MRA) can demonstrate multifocal intracranial arterial stenoses and a “beading” appearance of small- to medium-caliber vessels [8]. Vessel-wall imaging adds important complementary information, typically showing concentric thickening and enhancement of the affected arterial wall, beyond luminal abnormalities detected with conventional angiographic techniques. This vessel characterization provides information on inflammatory activity, helps distinguish vasculitis from angiographic mimics such as reversible cerebral vasoconstriction syndrome or atherosclerosis, and improves correlation with histopathological findings [9]. Digital subtraction angiography (DSA) usually reveals findings similar to those of MRA, such as multifocal stenoses [8], although its reported sensitivity is variable, ranging between 40 and 90%, and remains poorly defined [10]. Our study is limited by the lack of MRA, DSA, or VWI at presentation. This constraint is representative of a common diagnostic pitfall: when a parenchymal inflammatory syndrome is suspected, the vascular tree is often under-investigated. Given that VWI can distinguish inflammatory vasculitis by showing concentric enhancement, its absence in our cases likely delayed the definitive diagnosis of PACNS until histopathological re-evaluation was necessitated by clinical relapses.

In contrast, the radiological spectrum of SLIPPERS is less well characterized (Table 3). Described features include an angiocentric distribution of lesions with intense gadolinium enhancement [11]. The involvement is predominantly or exclusively supratentorial, with nodular foci (>3 mm) that are hyperintense on T2/FLAIR sequences [11]. Thus, while there is significant overlap between the MRI findings of SLIPPERS and PACNS, advanced angiographic techniques (MRA, vessel-wall imaging, and DSA) may provide additional clues favoring a diagnosis of vasculitis.

Clinically, both entities may present with variable and nonspecific symptoms, including focal neurological deficits, seizures, headache, or cognitive impairment [12]. Similarly, their therapeutic response is comparable: treatment consists of induction of remission with high-dose glucocorticoids, followed by long-term immunosuppression using cytotoxic agents such as cyclophosphamide or methotrexate to prevent relapses [13].

Histopathological findings remain central to the distinction between the two conditions. In PACNS, brain biopsy demonstrates transmural vascular inflammation involving the full thickness of the vessel wall [2,3]. However, the focal and segmental distribution of vasculitic lesions may lead to sampling error, producing false negatives in up to 50% of cases [14]. Several histopathological subtypes of PACNS have been described, with the lymphocytic variant being the second most common, after the granulomatous pattern. The former subtype is characterized by a predominantly lymphocytic inflammatory infiltrate, with few plasma cells [3], and is typically composed of CD8+ T cells, sometimes mixed with B cells and macrophages [15]. As a reactive process, monoclonality is possible. In this patient, the clonal signal was interpreted as an exaggerated immune response with a dominant specificity, a phenomenon documented in chronic inflammatory conditions where a limited number of T-cell clones expand in response to a persistent antigen [16]. By contrast, in SLIPPERS, biopsy also shows a dense lymphocytic infiltrate, but in this case, the distribution is predominantly perivascular, accompanied by diffuse parenchymal involvement. Both gray and white matter can be affected, and the infiltrate is largely composed of T-lymphocytes, particularly of the CD4+ subtype [17]. The most notable histological distinction, therefore, lies in the distribution of the lymphocytic infiltrate: perivascular in SLIPPERS versus transmural in PACNS. Nonetheless, differentiation is often difficult because biopsy material is usually limited, and vasculitic changes of PACNS used to be patchy (Table 4).

Initially, both patients were treated with a methotrexate-based steroid-sparing regimen, following established guidelines for relapsing CLIPPERS phenotypes [18]. In these parenchymal inflammatory syndromes, methotrexate is considered a first-line maintenance agent for sustaining a relapse-free state when doses are maintained. However, the therapeutic strategy was escalated in Case 2 following radiological recurrence while on methotrexate. The identification of transmural vasculitis and the lack of sustained response to methotrexate prompted the use of cyclophosphamide, which is the standard induction agent for aggressive or medium-vessel PACNS [3].

**Table 3:** Summary of the radiological and histopathological findings of SLIPPERS cases reported in the literature.

Case	Age/Sex	Radiological Findings			Histopathological Findings	
		Location	T2-FLAIR	Contrast Enhancment	Distribution	Lymphocyte Subtype
Armand 2015 [1]	-	Supratentorial	Hyperintense lesions similar to CLIPPERS	Yes	Perivascular inflammation	CD4/CD8
Armand 2015 [1]	-	Supratentorial	Hyperintense lesions similar to CLIPPERS	Yes	Perivascular lymphocytic inflammation	CD3
Horng 2017 [19]	56/M	Diffuse periventricular and deep white matter, amygdala, and hippocampus	Hyperintense lesions	Perivascular puntiform enhancement.	Perivascular lymphocytic inflammation	CD4/CD8+ and CD20, CD68
Sudhakar 2021 [20]	71/F	Right occipital lobe, right precentral gyrus, and multiple smaller lesions in the right periventricular region	Hyperintense lesions, vasogenic edema	Nodular enhancement	Perivascular lymphocytic inflammation	CD4 > CD8, Foamy macrophages and scattered small B cells.
Picarelli 2021 [21]	39/F	Subcortical and periventricular white matter lesions predominantly involving the right frontoparietal lobe and insula	Hyperintense lesions, vasogenic edema without mass effect	Perivascular puntiform and curvilinear enhancement.	Perivascular and leptomeningeal lymphocytic inflammation	CD4, CD8, CD20 and CD 86
Vattoth 2022 [11]/Khan 2023 [22]	21/M	Right parieto-occipital and periventricular white matter, involving the right side of the splenium of the corpus callosum	Hyperintense lesions	Patchy nodular and curvilinear enhancement	Perivascular lymphocytic inflammation	CD3, CD4, CD8 Foamy macrophages and scattered small B cells
Freua 2023 [17]	34/F	Bilateral frontal subcortical white matter	Hyperintense lesions	Perivascular puntiform and curvilinear enhancement	Perivascular lymphocytic inflammation	CD3, CD68
Tsibonakis 2023 [23]	70/F	Bilateral frontotemporal and periventricular white matter.	Hyperintense lesions	Perivascular nodular enhancement	Perivascular and gray matter lymphocytic inflammation	CD4+ > CD8+
Acir 2025 [24]	26/F	Left frontal and parietal lobes	Hyperintense lesions	Linear-patchy enhancement	-	-
Saini 2025 [25]	57/M	Bilateral frontal lobes, anterior temporal lobes, external capsule, right thalamus, right corona radiata with extension to anterior corpus callosum	Hyperintense lesions	Peppering contrast enhancement	Perivascular lymphocytic inflammation, predominantly in the basal region of the cortex and cortical-subcortex junction	CD3, CD4 and CD68
Mirg 2025 [26]	31/M	Bilateral cerebral hemispheres, basal ganglia, thalami, brainstem, and cerebellum Long segment intramedullary cervical cord lesion	Hyperintense lesions	Linear and nodular enhancement	-	-

M: Male; F: Female.

**Table 4:** Summary of characteristics of SLIPPERS and PACNS.

	SLIPPERS	PACNS
Anatomical Distribution	Strictly supratentorial	Whole CNS
T2/FLAIR	Hyperintense pseudonodular foci (>3 mm) with angiocentric distribution	Multifocal hyperintensities, often bilateral, cortical or subcortical
Gadolinium Enhancement	Characteristic and intense pseudonodular, punctate, or “peppering” enhancement.	Patchy parenchymal, nodular or leptomeningeal enhancement.
Angiography (DSA/MRA)	Typically normal	Segmental stenoses (“beading”)
SWI	Normal	microhemorrhages
Vessel-Wall Imaging	Not described	Concentric wall thickening/enhancement
Histopathology: Distribution	Predominantly perivascular lymphocytic sleeves.	Transmural inflammation (invasion of the full thickness of the vessel wall).
Histopathology	Dense lymphocytic (CD4+ T) infiltrate with predominantly perivascular distribution, without transmural inflammation	Transmural vascular inflammation involving the full thickness of the vessel wall, with predominantly CD8+ T cells

## 4. Conclusions

The current body of evidence regarding SLIPPERS is still scarce and does not yet provide sufficient robustness to define it as a distinct nosological entity. Given the broad clinical, radiological, and histological similarities with PACNS, differential diagnosis remains highly challenging. We therefore suggest that in patients with a suspicion of SLIPPERS, it is essential to carefully review MRI findings—particularly angiographic and vessel-wall imaging sequences—and to critically assess the pattern of lymphocytic infiltration on biopsy, both of which may strongly suggest an underlying diagnosis of PACNS.

## Figures and Tables

**Table 2:** Systematic mapping of clinical cases against established PACNS diagnostic criteria: Calabrese and Mallek [4]/Birnbaum and Hellmann [5].

Diagnostic Criteria	Case 1	Case 2
Unexplained neurological deficit	Seizures, memory impairment, and facial neuralgia.	Recurrent focal-to-generalized seizures.
Histopathological evidence of vasculitis	Re-evaluation revealed transmural lymphocytic infiltrate.	Transmural T-cell infiltrates (elastic stain confirmation).
Exclusion of systemic mimics	Negative systemic workup (CT, PET, serologies, autoimmune panels).	Negative systemic workup.
Diagnostic certainty level	Definite PACNS (tissue biopsy confirmation).	Definite PACNS (tissue biopsy confirmation).
MRI findings	Hyperintense T2/FLAIR lesions, with nodular enhancement.	Multifocal nodular enhancement and microhemorrhages (SWI).
CSF profile	Normal	Mild hyperproteinorrachia (79 mg/dL).

## Data Availability

The original data presented in the study are included in the article, further inquiries can be directed to the corresponding author.
